# High‐Contrast Optical Modulation from Strain‐Induced Nanogaps at 3D Heterogeneous Interfaces

**DOI:** 10.1002/advs.201903708

**Published:** 2020-04-26

**Authors:** Donghwi Cho, Young‐Seok Shim, Jae‐Wook Jung, Sang‐Hyeon Nam, Seokhwan Min, Sang‐Eon Lee, Youngjin Ham, Kwangjae Lee, Junyong Park, Jonghwa Shin, Jung‐Wuk Hong, Seokwoo Jeon

**Affiliations:** ^1^ Department of Materials Science and Engineering KAIST Institute for the NanoCentury Korea Advanced Institute of Science and Technology (KAIST) Daejeon 34141 Republic of Korea; ^2^ Division of Materials Science & Engineering Silla University 140 Baegyang‐daero 700beon‐gil Sasang‐gu Busan Korea; ^3^ Department of Civil and Environmental Engineering Korea Advanced Institute of Science and Technology (KAIST) Daejeon 34141 Republic of Korea; ^4^ Structural Safety & Prognosis Research Division Korea Atomic Energy Research Institute (KAERI) Daedeok‐daero 989‐111 Yusung‐gu Daejeon 34057 South Korea; ^5^ Department of Information Security Engineering Sang Myung University Cheonan‐si Chungcheongnam‐do 31066 Republic of Korea; ^6^ School of Materials Science and Engineering Kumoh National Institute of Technology Gumi Gyeongbuk 39177 Republic of Korea

**Keywords:** 3D nanostructures, air gaps, scatterers, smart windows, stretchable nanocomposites

## Abstract

The realization of high‐contrast modulation in optically transparent media is of great significance for emerging mechano‐responsive smart windows. However, no study has provided fundamental strategies for maximizing light scattering during mechanical deformations. Here, a new type of 3D nanocomposite film consisting of an ultrathin (≈60 nm) Al_2_O_3_ nanoshell inserted between the elastomers in a periodic 3D nanonetwork is proposed. Regardless of the stretching direction, numerous light‐scattering nanogaps (corresponding to the porosity of up to ≈37.4 vol%) form at the interfaces of Al_2_O_3_ and the elastomers under stretching. This results in the gradual modulation of transmission from ≈90% to 16% at visible wavelengths and does not degrade with repeated stretching/releasing over more than 10 000 cycles. The underlying physics is precisely predicted by finite element analysis of the unit cells. As a proof of concept, a mobile‐app‐enabled smart window device for Internet of Things applications is realized using the proposed 3D nanocomposite with successful expansion to the 3 × 3 in. scale.

## Graphical abstract

Mechano‐responsive smart windows are developed using stretchable 3D hybrid nanocomposites. The inserted periodic oxide nanoshell in the elastomer offers formation of light scattering air gaps upon stretching, and this provides high‐contrast optical modulation. It is demonstrated that smart window devices can be prepared for self‐responding to the surrounding environments and temporary projection screens by integrating with Internet of Things technology.

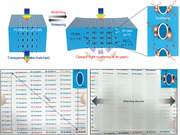

Over the past decades, there have been numerous efforts to develop climate‐adaptive building shells, particularly “smart windows”, to solve problems related to global energy consumption and simultaneously exert a positive impact on the indoor environmental quality of buildings.^[^ ^1^, ^2^, ^3^, ^4^ ^]^ To this end, various types of smart windows based on electrochromic,^[^ ^5^, ^6^ ^]^ thermochromic,^[^ ^7^, ^8^ ^]^ and photochromic^[^ ^9^ ^]^ materials have been developed that undergo external‐stimulus‐triggered changes in their chemistry and/or morphology, thus enabling control of their transmittance by modulating their coloration. Despite the active optical modulation capabilities of these materials, some drawbacks, such as complex fabrication steps, long response times, unnecessary coloration, excessive power consumption, low thermal resistance, and degradation of organic‐based chromophores or dyes, have not yet been resolved.^[^ ^10^, ^11^ ^]^ These issues may be overcome by means of an optical modulation scheme based on structural modification in response to mechanical deformation.

The well‐known principle of the mechanical modulation of optical transmission is to use a pretexturized elastomer surface that can be flattened by an external force. For example, mechano‐responsive smart windows (MSWs) can be created by treating prestrained poly(dimethylsiloxane) (PDMS) with oxygen plasma or ozone to form surface wrinkle patterns or by imprinting micro/nanopillar arrays on such surfaces.^[^ ^12^, ^13^, ^14^, ^15^, ^16^ ^]^ An intrinsically opaque surface‐textured elastomer film becomes transparent upon stretching and reversibly returns to its original state upon release. While this approach has the advantage of being applicable to most commercial elastomers, the direction of deformation is limited to an in‐plane direction (uniaxial/biaxial), and the modulation contrast achieved based on the surface texture alone is generally low (<46%).^[^ ^13^, ^14^, ^15^, ^16^ ^]^ It is noted that the optical modulation mentioned in these studies is normal transmittance change, which is generally induced by the light scattering rather than light absorbing. Additionally, the scattering interfaces are exposed and are not subject to any passivation; consequently, they can be vulnerable to external damage and lose their function in an index‐matched medium (e.g., in water).^[^ ^14^ ^]^

Recently, a hybrid nanocomposite consisting of silica nanoparticles randomly dispersed in a PDMS matrix has been employed to develop a 3D geometric MSW.^[^ ^2^, ^17^, ^18^ ^]^ Compared with surface‐textured elastomeric films, this 3D hybrid nanocomposite film exhibits a significantly improved optical density due to the simultaneous formation of numerous air gaps at the interfaces between the silica nanoparticles and the PDMS during stretching. Since there is a plurality of scattering layers in the travel direction of the light, high optical modulation (≈70%) can be achieved at a low global strain level (≈100% strain).^[^ ^2^ ^]^ Nonetheless, an in‐depth study of the underlying physics of the governing factors, namely, the effective refractive index and the separation distance between neighboring phases, is lacking, and how to improve the scattering performance remains unclear. Given the random boundaries of the scattering structures, constructing a structural unit cell model for quantitative analysis is limited. In addition, a high core volume of the particles leads to decreased transmittance of the initial transparent state, an increase in weight, degradation in flexibility, and a limited number of scattering sites. Therefore, it is imperative to develop a more controlled approach that enables the realization of high‐performance MSWs and practical demonstrations, unlike previous efforts that have focused only on suggesting methodologies and investigating their optical switching performances.

Here, we report a new type of 3D hybrid nanocomposite film with a periodic network structure that allows quantitative control of the embedded scatterers generated under tensile strain. The key strategy is to optimize a thin (≈13 µm) but optically dense 3D scattering structure by incorporating an Al_2_O_3_ nanoshell (≈60 nm) between the highly periodic elastomers in a 3D nanonetwork. Thus, high performance can be achieved, including a giant transmission modulation of up to 74% at visible wavelengths (from 90% initial transmission to 16% in the scattering state under strain) and superb durability over more than 10 000 cycles of 40% stretching/releasing. Due to the uniform geometric structure, it is possible to clarify the underlying mechanism, namely, the reversible formation/recovery of artificial light scattering zones (ASZs) in the stretched/released film, via finite element analysis for the first time. As demonstrated by a proof of concept of an active component for potential home automation within the Internet of Things (IoT) paradigm, new classes of smart window devices that can self‐regulate their behavior in a user‐friendly manner are possible and promising for future use in various applications that require active transmission modulation.

When binary phases with index‐mismatched boundaries (e.g., particles, pores, or grain boundaries) exist in an optically transparent medium, the medium generally becomes opaque by scattering broadband visible light in all directions. According to light scattering theory, the dimensionless size parameter (*χ*) in the scattering regime can be simply expressed as

(1) $$\begin{array}{c} \chi \;=\frac{2\pi r}{\lambda }\; \end{array}$$

where *r* is the radius of a spherical scattering phase and *λ* is the wavelength of the incident light. Mie scattering, which is related to the optical modulation based on light scattering effect, occurs at *χ* ≈ 1, that is, when *r* is roughly comparable to *λ*/2*π* rather than much smaller or much larger.^[^ ^19^, ^20^ ^]^ Meanwhile, if the two phases are in conformal contact with each other, then there is no scattering effect except for negligible reflection at the interface due to the continuous boundary (**Figure** **1**a[i]).^[^ ^21^ ^]^ However, as the air gap between the two phases increases to become no longer negligible but still smaller than *λ*/2*π*, scattering occurs at the air gap, which behaves like a single boundary (Figure 1a[ii]). As the distance becomes comparable to or larger than *λ*/2*π*, the light scattering becomes more active because both surfaces of the air gap/phase boundary behave as independent scattering boundaries (Figure 1a[iii]). Thus, the total scattering efficiency from these two successive boundaries becomes more significant than that in the single‐boundary case due to the cascade effect.^[^ ^21^ ^]^ Interestingly, this optical phenomenon can be dramatically enhanced by the synergetic interactions of scattered and diffuse light at various angles in the case of a complicated 3D scattering phase with multiple boundaries (Figure 1a[iv]).^[^ ^2^, ^17^, ^18^ ^]^ Thus, it is obvious that simultaneous manipulation of such 3D index‐mismatched boundaries offers an important means of adjusting the light transmission through the medium and therefore can be a powerful strategy for realizing high‐contrast optical modulation.

**Figure 1:**
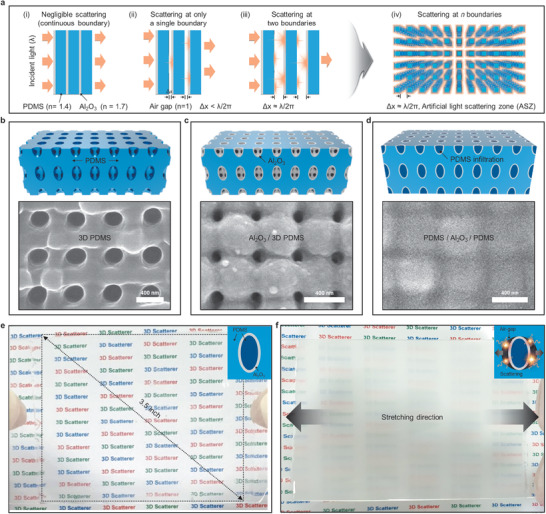
Design concept of and fabrication procedures for the 3D scatterer. a) Schematic illustrations showing how the light scattering effect depends on the distance between neighboring boundaries. b) Preparation of the 3D PDMS, c) the Al_2_O_3_‐nanoshell‐coated 3D PDMS, and d) the structure after the infiltration of PDMS into the pores of (c). e) Digital photographs of the large‐area 3D scatterer before stretching (transparent state) and f) after stretching (opaque state). Inset: the air gap formation/recovery process in the 3D scatterer upon stretching/releasing.

We propose a 3D scatterer fabricated by inserting an ultrathin oxide nanoshell into a 3D elastomer nanonetwork to control the 3D heterogeneous interfaces. The resulting mechanical deformation generates countless air gaps, which act as ASZs, facilitated by the forced shaping of the high‐modulus oxide. If an elastomer is placed against another elastomer of the same modulus without such a heterojunction, the two different elastomers, will not generate a strain‐induced air gap at the interface due to volume conservation even though the boundary is separable in principle. Likewise, when an elastomer is placed against another elastomer with a different modulus, only a tiny air gap can be generated, which will serve as a single boundary, as discussed above. By contrast, the optical density in a small unit cell can be maximized by introducing an oxide nanoshell, which has a higher refractive index and a markedly different elastic modulus compared to those of the elastomer. Thus, this nanoshell structure is key to realizing numerous pairs of index‐mismatched boundaries separated by air gaps (≈160 nm at 60% strain) to effectively scatter incident light at various angles. After release, the ASZs disappear due to the conformal restoration of the elastomer, causing the inherent interfaces to act as a continuous medium for the incident light.

Figure 1b–d illustrates the fabrication procedures for the proposed 3D scatterer. First, a 3D nanonetwork of PDMS (3D PDMS) is replicated from a 3D polymeric template prefabricated using a wafer‐scale 3D nanofabrication technique (Figure 1b).^[^ ^22^, ^23^, ^24^, ^25^ ^]^ Then, Al_2_O_3_ (≈60 nm) is conformally deposited on the surface of the 3D PDMS via atomic layer deposition (ALD) after its surface has been modified to be hydrophilic through UV/ozone treatment (Figure S1, Supporting Information).^[^ ^22^, ^26^, ^27^, ^28^, ^29^, ^30^, ^31^, ^32^ ^]^ The size reduction of the pores in the 3D nanostructure proves the successful coating of the PDMS with the Al_2_O_3_ (Figure 1c; Figure S2, Supporting Information). Then, infiltration of the same PDMS into the pores results in the ASZs containing, continuous boundaries of three phases (PDMS/Al_2_O_3_/PDMS), as shown in Figure 1d. Before the PDMS infiltration, the film is opaque due to light scattering at the air–Al_2_O_3_ interfaces (refractive indices of *n*_air_ ≈ 1 and *n*_alumina_ ≈ 1.7) (Figure S3, Supporting Information). However, the sample becomes highly transparent (≈90% at 600 nm) once the interstitial pores have been filled with index‐matched PDMS (*n*_PDMS_ ≈ 1.4) (Figure 1e). This transparent film becomes opaque again when tensile strain is applied due to the generation of ASZs that can cause light scattering (Figure 1f). This ASZ‐based scattering occurs uniformly over a large area (>8 in.^2^) (Figure S4, Supporting Information).

To maximize the optical density of the 3D scatterer, the effect of coupling of two critical factors on the performances, namely, the thickness of the Al_2_O_3_ nanoshell and the number of repeated layers in the periodic 3D nanostructure, must be carefully optimized. First, **Figure** **2**a shows the strain (*ε*)‐dependent change in the normal transmittance of the 3D scatterer as a function of the Al_2_O_3_ thickness from 20 to 80 nm.^[^ ^27^, ^28^ ^]^ The film thickness is fixed as 13 µm, which is the highest value in this material system, and this effect is discussed in Figure 2b. As the Al_2_O_3_ thickness increases up to 60 nm, the optical modulation (normal transmittance change Δ*T* = *T*_initial_−*T*_stretched_) is gradually enhanced until a saturation point is reached (≈74% at 60% strain) (Figure S5, Supporting Information). In case of the total transmittance, the optical modulation is near 33% upon stretching of 60%, as shown in Figure S6, Supporting Information. In other words, a higher effective refractive index of the scattering phase relative to the air causes higher scattering at the boundaries.^[^ ^27^ ^]^ However, for an 80 nm thickness of Al_2_O_3,_ which is regarded as the transition value between the oxide buckling regime and the bulk‐like property regime,^[^ ^27^, ^28^ ^]^ the optical modulation is slightly decreased due to the decrease in *T*_initial_ caused by the increased volume fraction of the Al_2_O_3_ as well as reduced ASZ formation. Although parts of the Al_2_O_3_ nanoshell break under a large strain, the resulting nanometer‐scale cracks in the oxide can be restored, returning the material to its initial seamless boundary condition, because the low mass of the thin oxide shell cannot overcome the van der Waals force with the PDMS, thus preventing total delamination from the PDMS (Figure S7, Supporting Information). These cracks in the oxide do not significantly degrade the performance of the 3D scatterer as an optical material under our experimental conditions due to their negligible size relative to the wavelength of the incident light, as proven by the superb repeatability of the optical modulation. Compared with previous work using randomly dispersed oxide particles in an elastomeric composite (with a Δ*T* of 70% at a strain of 100%),^[^ ^2^ ^]^ the strain level needed to achieve the maximum Δ*T* with the same volume fraction of nanopores (a porosity of approximately 40%) is 66% smaller for the material presented in this paper (with a Δ*T* of 74% at a strain of 60%). It is particularly noteworthy that the periodic structure ensures the effective formation of the ASZs by enforcing a regular stress distribution throughout the structure. To fully utilize the scattering effect of the repeated layers in the 3D nanostructure (with a periodicity of ≈1.36 µm in the direction perpendicular to the surface), 3D scatterers with 3D nanostructures of different thicknesses were prepared using negative‐tone photoresists (Figure S8, Supporting Information).^[^ ^22^, ^23^ ^]^ The 13‐µm‐thick sample, with 9 scattering layers, showed the widest range of optical modulation under our experimental conditions (Figure 2b). The ratio of the thickness of the 3D layer to that of the bulk supporting layer (fixed at ≈500 µm) was varied from 0.010 to 0.025 in three colors (red, green, and blue) covering the visible wavelength range, and the specimens of each color exhibited a linear increase in optical density with an increasing thickness ratio (Figure 2c). This linearity can be advantageous for quantifying and controlling the transmittance in MSW applications.

**Figure 2:**
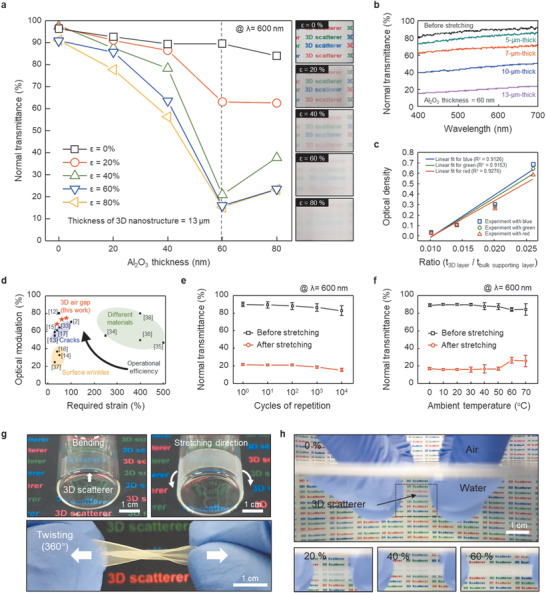
Strain‐dependent optical properties of the 3D scatterer. a) Normal transmittance spectra of 3D scatterers with different Al_2_O_3_ thicknesses at a wavelength of 600 nm as a function of the tensile strain (*ε*). b) Normal transmittance versus wavelength under a tensile strain of 40% for various 3D scatterers possessing different numbers of scattering layers, corresponding to different thicknesses, with a fixed Al_2_O_3_ deposition thickness of 60 nm. c) Comparison of optical density for different ratios of the thickness of the 3D layer to that of the bulk supporting layer. d) Optical modulation performances of recently reported MSWs. e) Performance of the 3D scatterer under cyclic 40% stretching and releasing. f) Optical modulation at various temperatures. g) Digital photographs of wrapped and twisted 3D scatterers. h) Digital photographs of the 3D scatterer under water‐immersed conditions.

For further application in practical MSWs, it is highly desirable to achieve high‐contrast optical modulation at a small strain and with high cyclability and durability. The levels of optical modulation are plotted versus the required strains in Figure 2d for previously reported MSWs.^[^ ^2^, ^12^, ^13^, ^14^, ^15^, ^16^, ^17^, ^33^, ^34^, ^35^, ^36^, ^37^, ^38^ ^]^ For an efficient MSW, the performance should lie in the upper left part of the plot. We achieve competitive performance by utilizing a relatively small strain to induce a large Δ*T* due to the optimization of the scattering phases. In addition, the exceptional optical modulation of our material persists over a very large number (>10 000) of repeated 40% stretching/releasing cycles without severe degradation (Figure 2e; Figure S9, Supporting Information). Consider a car window application on a scorching day, when temperatures could rapidly rise above 40 °C smart windows for such an application should operate reliably even in hot environments.^[^ ^17^ ^]^ For testing under similar conditions, our films were placed on hot plates for 12 h. Interestingly, the 3D scatterers could endure even under elevated temperatures due to their stable material properties (Figure 2f).^[^ ^39^ ^]^ Except for the slight deviation at 60 °C due to the thermal‐dependent mechanical properties of PDMS,^[^ ^23^ ^]^ it was possible to achieve such stability which can be a technical challenge for conventional chromic materials because of their low thermal resistance.^[^ ^5^ ^]^

Figure 2g demonstrates the superb flexibility of our 3D scatterer. The thin 3D scatterer can be compliantly wrapped onto the curved surface of a vial, which has bending radius of 1.5 cm, while accommodating the bending‐induced strain due to its superior conformality. The stretched opaque film can also be conformally attached to the curved vial surface. The deformability of the 3D scatterer is not limited to bending; it also can be twisted through 360°. In addition, it successfully exhibits optical modulation in water because the ASZs are protected from unintentional index matching with the surrounding medium (Figure 2h; Movie S1, Supporting Information). This intriguing water‐proof feature of our film is attractive for a wide range of MSW applications, including privacy protection in swimming pools and water sports equipment.^[^ ^40^ ^]^ However, due to the PDMS swelling effect, this operation will be limited in the exposure of the organic solvents.^[^ ^22^ ^]^ Nevertheless, it can be another way to detect the target solvent by tuning optical properties and can be one of the promising further works.

The overall scattering mechanism can be controlled by means of the periodic structure of the 3D scatterer. The unique ASZ formation at the heterogeneous interfaces was verified through the use of 3D unit cell models in the LS‐DYNA software (Livermore Software Technology Corporation), as shown in **Figure** **3**a. The optimal sample parameters identified from Figure 2a were selected for this purpose (60‐nm‐thick Al_2_O_3_ and a strain of <60%). Air gaps were found to be continuously formed on both sides of the center ellipsoid and the bridge elements of each 3D unit cell. The volume fraction of the generated air gaps was enhanced from 16.55 to 37.46 vol% as the applied strain was increased from 20% to 60%. It has been reported that 3D nanostructured Al_2_O_3_ (with a thickness of 55 nm) itself shows a mechanical engineering limit of approximately 15% strain before the occurrence of cracking and rapid collapse of the whole structure.^[^ ^28^ ^]^ However, as discussed in regard to Figure 2a, the nanocracks in the oxide on the PDMS matrix caused by further stretching may be restored because the oxide does not fully delaminate due to the van der Waals force with the PDMS. The resultant interconnected air gaps are represented as three different sizes: the largest air gap is located at the center ellipsoid (gap 1), and the gaps at the bridge elements are of two different sizes (the intermediately sized gap is gap 2, and the smallest is gap 3). Due to the uniform stress distribution throughout the 3D scatterer, these air gaps linearly expand under continuous stretching (Figure 3b; Figure S10, Supporting Information). Based on the dependence of the light scattering behavior on the separation distance, as depicted in Figure 1a, the transition region between the region in which the 1‐scattering‐boundary behavior is dominant (≈60 nm) and the region in which the 2‐scattering‐boundaries behavior is dominant (≈120 nm) can be roughly defined by considering the wavelength range of the visible light. This calculated tendency is in good agreement with the experimental results presented in Figure 2a. As the strain level increases from approximately 20% to 40%, the volume fraction of gap 1 increases beyond the 1‐boundary‐dominant condition, indicating more active scattering, which leads to a significant normal transmittance drop. When the strain is greater than 40%, gap 2 additionally contributes to the scattering in the 2‐boundaries‐dominant condition, and the optical modulation is improved.

**Figure 3:**
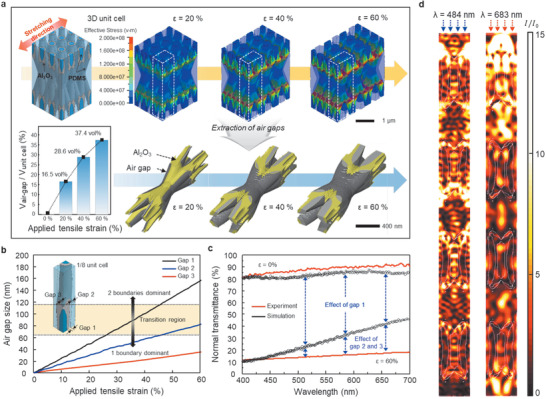
Mechanical and optical simulations of the 3D scatterer. a) Contours of the von Mises stress in 9 unit cells of the 3D scatterer with an Al_2_O_3_ coating thickness of 60 nm under strains of 20%, 40%, and 60%. The white dotted lines enclose a single unit cell. The air gap models were extracted from a single unit cell of the 3D scatterer at each applied tensile strain level. b) Plot of the generated air gap sizes under various tensile strains. c) Comparison of the experimental and simulated results before and after stretching under a strain of 60%. d) Color maps of the E‐field intensity distribution in the cases of 484‐nm and 683‐nm light passing through the 3D scatterer.

To interpret the optical behavior of the air gaps in ASZ terms, the largest gap, that is, gap 1, which plays a critical role in the transmittance drop as the first scattering phase to reach the 2‐boundaries‐dominant condition, was chosen from among the three types of air gaps as the basis for a simplified model to be used in a finite‐element‐based optical analysis.^[^ ^23^, ^24^, ^25^ ^]^ Although the optical calculation of the smart windows with finite‐difference time‐domain (FDTD) have been studied,^[^ ^16^, ^41^, ^42^ ^]^ to the best of our knowledge, this multiphysical analysis, presented below, is the first to combine mechanics and optics to verify the MSW mechanism. The simulated normal transmittance after stretching under a strain of 60% gradually decreases as the wavelength becomes shorter due to the grating effect of the periodic structure.^[^ ^43^ ^]^ However, the experimental results exhibit a rather constant and improved modulation contrast due to the significant enhancement of the scattering from the additional scattering phases: the finely controlled gaps 2 and 3 and the intrinsic surface wrinkle formation of the elastomeric substrate.^[^ ^2^, ^17^, ^18^ ^]^ Figure 3d presents direct evidence of ASZ formation at the 3D heterogeneous interfaces in the form of the electromagnetic field (E‐field) distribution over the cross section at the center of the 3D scatterer. For both a short wavelength (484 nm) and a long wavelength (683 nm), the clear localization of the E‐field at the boundaries proves the active light scattering effect. Overall, the 3D scatterer can serve as an ideal structure for inducing active light scattering (Figure S11, Supporting Information).

The key to optimize the ASZs is to introduce different mechanical/optical properties of the matrix and nanoshell. These mechanical differences, including the elastic modulus and bonding strength,^[^ ^44^, ^45^ ^]^ lead to displacement at the interfaces; thus, the refractive index mismatches at the heterogeneous interfaces give rise to effective reflection sites (PDMS/air and air/Al_2_O_3_). Through these behaviors, the small volume fraction of Al_2_O_3_ used to construct the nanoshell structure ensures not only a high initial transmittance but also ASZ formation under stretching. Therefore, the investigation of other material systems comprising a transparent nanoshell with a higher refractive index and a low bonding strength with the adjacent matrix is another promising direction for future work.

It is obvious that the IoT paradigm allows smart windows to autonomously respond to their surrounding environments and promises to fuel a revolution in home automation. In smart windows developed for IoT applications, low power consumption, ease of integration with electronic circuits, and the ability to communicate with sensors are required.^[^ ^46^, ^47^, ^48^, ^49^ ^]^ Here, we first demonstrate a proof of concept of a compelling application for such a class of smart devices. **Figure** **4**a shows a block diagram of an interface circuit comprising two digital servomotors, an ambient light sensor, and a wireless communication module. An overview of our device, which acquires surrounding illumination data from the sensor and subsequently exhibits an optical modulation response depending on the operation mode—remote manual control through a connected mobile app or automatic response mode—is shown in Figure 4b. In accordance with a user‐defined threshold value of ambient illumination, the MSW becomes opaque to block or reflect light under conditions of excessive illumination and returns to a transparent state under desirable lighting conditions for daily life in the room (Figure 4c). Based on the 3D scatterer's behavior as a single cohesive stretchable object, the transmittance of the window can be adjusted promptly and automatically within approximately 1 s (Movie S2, Supporting Information). Moreover, since the servomotors activate only when controlling the angle, the static current is zero until the next command.

**Figure 4:**
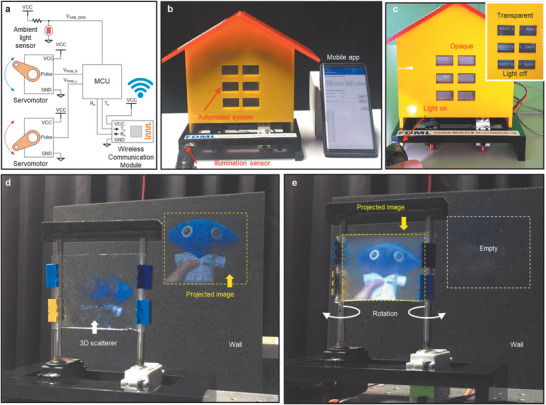
Demonstrations of IoT applications: a self‐regulating MSW and a beam projection screen. a) A circuit diagram and b) an overview of a mobile‐app‐enabled smart window device. c) Automatic response mode of the MSW, in which it responds to the surrounding illumination conditions. d) The off state (transparent) and e) the on state (opaque) of the proposed 3D scatterer operating as a projection screen.

Another possible use of the proposed 3D scatterer is as a temporary projection screen that can focus a beam‐projected image as a sort of aesthetic wallpaper. In this intriguing application, the common drawbacks of conventional projection screens, such as the spatial constraints for installations consisting of a screen and a supporting mainframe and the time required to pull out the screen scroll, are significantly reduced. Figure 4d,e presents the screen‐off and screen‐on modes, respectively, demonstrating that the projected image is clearly focused on either the wall, because the film is transparent except for the intrinsic reflection from the PDMS, or the film (Figure S12, Supporting Information). It should be noted that this screen application benefits from the exceptional scalability of the 3D scatterer. Consequently, this kind of wall‐type display, representing an emerging stretchable or rollable display, might represent a promising breakthrough as a replacement for conventional large, rigid displays that could meet the aesthetic needs of various architectural scenarios.

The realization of high‐contrast optical modulation is of fundamental importance to many fields; in addition to MSWs, the potential applications include privacy or security applications, agricultural and biological applications. Therefore, the precise control of the formation of ASZs at 3D heterogeneous interfaces in the thin, but optically dense, periodic 3D nanocomposite proposed here can serve as the basis for a new and powerful technology for constructing on‐demand architectures with exceptional performance. A critical component of this work is the theoretical prediction and experimental confirmation of the characteristics of the strain‐induced nanogaps, enabling the maximization of the light scattering to achieve high‐contrast normal transmittance modulation. Of equal importance is the demonstration of an IoT application achieved by integrating an inch‐scale MSW (>8 in.^2^) into a mobile‐app‐enabled smart window device as an active component. Exploring other advanced materials that exhibit the same fundamental physics offers the potential to realize new classes of windows endowed with artificial intelligence in the future.

## Experimental Section

##### Preparation of the 3D Polymeric Template

The 3D PDMS film was prepared via proximity‐field nanopatterning (PnP) and material conversion procedures.^[^ ^22^, ^23^, ^24^, ^25^, ^26^, ^27^, ^28^, ^29^, ^30^, ^31^, ^32^ ^]^ The fabrication method for the phase mask and the detailed process of the PnP technique had been described in previous works.^[^ ^22^, ^23^, ^24^, ^25^, ^26^, ^27^, ^28^, ^29^, ^30^, ^31^, ^32^ ^]^ In brief, a 3D polymeric template was first prepared for refilling of PDMS elastomer (Sylgard 184, Dow Corning) into the photoresist mould. A thin layer of photoresist (≈100 nm, NR7‐80p, Futurrex, Inc.) was spin coated onto a plasma‐treated substrate as a sacrificial layer. The substrate was plasma treated to improve the coverage and uniformity of the photoresist. After hard baking of the substrate, a relatively thick layer (≈15 µm, NR5‐6000p, Futurrex, Inc.) of photoresist was spin coated onto the sacrificial layer. Then, the substrate was brought into soft contact with a conformal phase mask patterned with a square array of holes (with a diameter of ≈480 nm, a depth of ≈400 nm, and a periodicity of ≈600 nm).^[^ ^22^, ^23^, ^24^, ^26^, ^27^, ^28^, ^29^, ^30^, ^31^, ^32^ ^]^ After the 3D nanofabrication process, general lithographic procedures, including a postbaking step, developing (RD6, Futurrex, Inc.), and a rinsing step with deionized water, were performed to complete the fabrication of the 3D polymeric template.

##### Preparation of 3D PDMS for the Stretchable Scaffold

To prepare the pores of the template for infiltration with PDMS prepolymer, the template was treated with plasma (CUTE‐MP, Femto Science). Premixed PDMS with a weight ratio of 10 to 1 (monomer:crosslinking agent) was dispensed onto the 3D polymeric template.^[^ ^22^, ^23^ ^]^ Then, the overlayer on the template was precisely controlled by varying the speed of the spin‐casting procedure from 500 to 2000 rpm.^[^ ^22^, ^23^ ^]^ A degassing process in a desiccator guaranteed perfect infiltration of the PDMS prepolymer into the interconnected pores of the 3D template. After curing in an oven, the template and the sacrificial layer were cleanly removed with photoresist remover (RR41, Futurrex, Inc.). Finally, the pristine 3D nanostructured PDMS film was rinsed with deionized water.

##### Preparation of the 3D Scatterer

Since the optical modulation based on the silica‐like layer from UV/ozone treatment was not effective due to limited penetration depth,^[^ ^22^ ^]^ Al_2_O_3_ was chosen to coat the surface to induce high‐contrast performances. To achieve conformal coating of the Al_2_O_3_ nanoshell, the 3D PDMS was exposed to UV/ozone to make its surface hydrophilic, and the Al_2_O_3_ was carefully deposited via ALD (Scientech) at a low temperature of 90 °C.^[^ ^27^, ^28^ ^]^ An Al_2_O_3_ precursor and H_2_O as a reactant were used to prepare trimethyl aluminum (UP Chemical).^[^ ^27^, ^28^ ^]^ Commercial adhesive (3M tape) was attached to regions surrounding the 3D nanopattern to selectively expose the patterned region for further infiltration. Then, the nanocomposite was infiltrated with PDMS prepolymer with the same weight ratio as that of the 3D PDMS, namely, 10 to 1 (monomer:crosslinking agent), to perfectly fill the pores of the composite and match the refractive index. After a degassing process, the attached adhesive was removed, and the nanocomposite was cured in a 65 °C oven for 4 h.

##### Mechanical Modeling

LS‐DYNA, a nonlinear finite element analysis platform with a large library of material models and element formulations, was employed to perform mechanical simulations of the 3D scatterer (see the Supporting Information for details).

##### Optical Modeling

Optical simulations for calculating the scattering efficiency and its contribution to the transmittance drop were conducted using commercial FDTD simulation software (Lumerical) (see the Supporting Information for details).

##### Fabrication of a Mobile‐App‐Enabled Smart Window Device

Both the sides of the 3D scatterer were attached to two digital servomotors (DM‐BLS130TD, Doman RC) individually rotating in opposite directions to stretch/release the 3D scatterer by means of torque forces. To allow the device to respond to the surrounding illumination conditions, a commercially available cadmium sulfide (CdS) ambient light sensor and a Bluetooth module were incorporated into a single circuit.

##### Characterizations

The morphologies of the 3D nanocomposites were characterized using a field‐emission scanning electron microscope (S‐4800, Hitachi) operated at an accelerating voltage of 5–10 kV. Elemental mapping measurements were collected via energy dispersive spectroscopy (Magellan 400, FEI). The normal transmittance measurements were performed using a UV–vis spectrophotometer (SolidSpec‐3700, Shimadzu). To measure the normal transmittance under various strain conditions, the UV–vis spectrophotometer was coupled with a custom‐built stretcher. The total transmittance and reflectance were measured using the UV–vis spectrophotometer with an installed integrating sphere mode.^[^ ^12^, ^26^ ^]^ The stretched samples were placed at the entrance stage in the optical path of beam. After passing through the sample, all the scattered beams were collected by the photodetector. Cyclic tests and thermal stability tests were conducted with 40% stretched samples. All experimental results presented in the manuscript were reported as average values for many (>10) tested samples.

## Conflict of Interest

The authors declare no conflict of interest.

## Supporting information

Supporting Information

Supplemental Video 1

Supplemental Video 2
