# Supplementary material for: High‐Contrast Optical Modulation from Strain‐Induced Nanogaps at 3D Heterogeneous Interfaces
Source: Adv Sci (Weinh). 2020 Apr 26;7(11):1903708. doi: 10.1002/advs.201903708 (PMC7284194; doi:10.1002/advs.201903708)
Supplement: Supplementary file 1 — Supporting Information [file ADVS-7-1903708-s001.pdf]

Copyright WILEY-VCH Verlag GmbH & Co. KGaA, 69469 Weinheim, Germany, 2020.

Supporting Information

High-Contrast Optical Modulation from Strain-Induced Nanogaps at Three-Dimensional Heterogeneous Interfaces

Donghwi Cho[1,5], Young-Seok Shim[1,5], Jae-Wook Jung[2], Sang-Hyeon Nam[1], Seokhwan Min[1], Sang-Eon Lee[2], Youngjin Ham[1], Kwangjae Lee[3], Junyong Park[4], Jonghwa Shin[1], Jung-Wuk Hong[2], and Seokwoo Jeon[1*]*

Supporting Information

Mechanical modeling

The finite element method (FEM) is an effective methodology for solving problems with complicated geometries that are difficult to solve analytically. To solve such a problem, an object of interest is divided into small parts with simple shapes, which are called finite elements. The simple equations used to model these finite elements are combined to form a simultaneous equation that models the whole problem. The distributions of the physical quantities for the object are obtained by solving this simultaneous equation subject to the boundary conditions of the surface of each element.

The unit cells of the 3D scatterer were separately modeled as a PDMS component with body-centered tetragonal symmetry (BCT), an Al_2O_3 nanoshell component, and an infiltrated PDMS component that were assembled under contact conditions. Tetrahedral elements, which are the most versatile elements for modeling the arbitrary shapes of 3D structures,[1] were employed to discretize the complicated shapes of the 3D scatterer. Each interaction between components was described using contact conditions by means of the penalty method. This method is widely used for solving many contact problems in finite element analysis.[2] Plastic kinematic material models were used to describe the material properties of the PDMS and Al_2O_3. The material properties of the PDMS were defined as follows: a Young's modulus of 2.05 MPa, a Poisson's ratio of 0.499, and a yield stress of 700 kPa. For the Al_2O_3 elements, the Young's modulus, yield stress, and Poisson's ratio were assigned values of 150 GPa, 0.24, and 24.7 MPa, respectively, based on the properties of a nanolayer of Al_2O_3, which are given in reference.[3]

To alleviate boundary effects, we modeled a 3×3×3 structure, and the deformation of one cell in the modeled array was analyzed to represent the common response. For the discretization of the structure, 4,840,000 tetrahedral elements were used, and appropriate contact conditions

were imposed between components. All simulations were carried out using a workstation with a Dual Intel ® Xeon(R) CPU E5-2687W v4 @ 3.00 GHz with 48 threads and 256 GB of memory.

Optical modeling

We calculated the optical properties of the 3D scatterer using commercial finite-difference time-domain (FDTD) simulation software (Lumerical).[4,5] To analyze the proposed structure as a scatterer-dispersed medium, the unit cells for both 0% and 60% strain were truncated above and below their center region with a span of 400 nm, where the air gap (for 60% strain) is the thickest (gap 1). The truncated regions were then replaced with PDMS. The scattering cross sections and differential cross sections of the unit cells for 0% and 60% strain were then simulated via the software with the appropriate power monitors. A total-field scattered-field (TFSF) source was used to separate the incident field from the scattered fields. For the 60%-strained structure, the unit cell was anisotropic along the X and Z directions (where the Y direction lies along the long axis of the unit cell); therefore, simulations were performed separately for both X- and Z-polarized incident light.

The scattering cross section σ_{sca} is a quantitative measure of how much an object scatters incident light. It is defined as the ratio of the total scattered power P_{sca} (W) to the incident intensity I_{inc} (W/m^2) and thus has units of area (m^2).

$$\sigma_{sca} = \frac{P_{sca}}{I_{inc}}$$

The scattering cross section may be larger than, equal to, or smaller than the physical cross section and roughly represents the area over which the incident light is disturbed by the object. The differential scattering cross section (m^2/°) describes the distribution of the scattered power with respect to the polar angle θ, which is measured from the Y axis in this case. In general, the differential cross section also varies with the azimuthal angle ϕ, but for simplicity,

this variation was averaged out under the assumption of an equal probability of scattering for any angle ϕ. The differential cross section was determined in the FDTD software by detecting the far-field scattered power at each angle around the unit cell.

The scattering cross sections and differential cross sections from the FDTD software for the 0%-strained structure, the 60%-strained structure with X-polarized incident light, and the 60%-strained structure with Z-polarized incident light were then fed into a Monte Carlo simulation to determine the 0th-diffraction-order transmittance of the entire film for each case. A total of 1 million photons of random wavelengths in the visible region impinged on the film in the normal direction. The following factors were considered when calculating the photon trajectories:

a) Air-PDMS boundary reflection and transmission: The probabilities of reflection and tr ansmission at each boundary were determined using the Fresnel equations.

b) Frequency of scattering events: The average mean free path of the photons inside the f ilm was determined from the scattering cross section as follows:

$$\mathrm{mfp} = \frac{1}{\sigma_{sca}\rho_v}$$

where ρ_v is the volume density of scatterers (number of scatterers per unit volume). The actual path length for each individual photon was then randomly determined following an exponential distribution with the previously determined *mfp* as the mean. The probability density function for a path length of *x* is as follows:

$$f(x) = \frac{1}{\mathrm{mfp}} e^{-\frac{x}{\mathrm{mfp}}} \qquad x \geq 0$$

c) Direction of scattering: The direction of the photon's path after each scattering event was determined based on the differential scattering cross section. The probability of sc attering at an arbitrary polar angle θ was set to be proportional to the differential cross

section at that angle.

After each photon exited the simulation region, it was determined whether that photon was reflected or transmitted and at which angle. This enabled the calculation of the transmittance as a function of the exit angle, which was measured from the axis normal to the plane of the film. The transmittance for the 60%-strained structure was calculated as the average of the transmittances for X-polarized and Z-polarized incident light.

Supporting figures

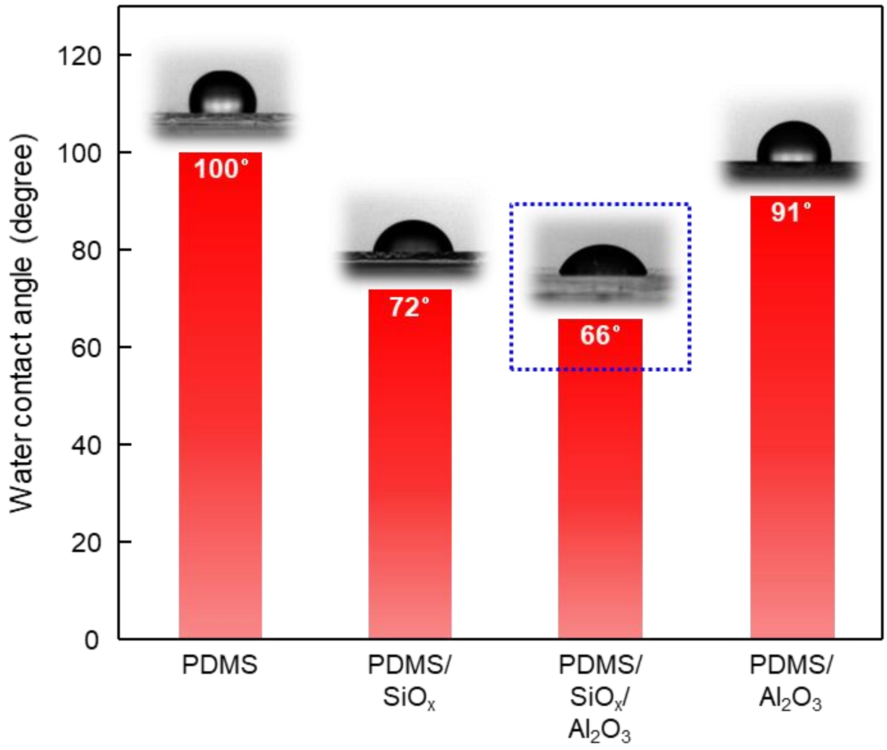

Figure S1. Water contact angles of various PDMS films. The surface modification was to control the surface from hydrophobic to hydrophilic. The few SiO_x layer was generated by UV/Ozone treatment of the PDMS.

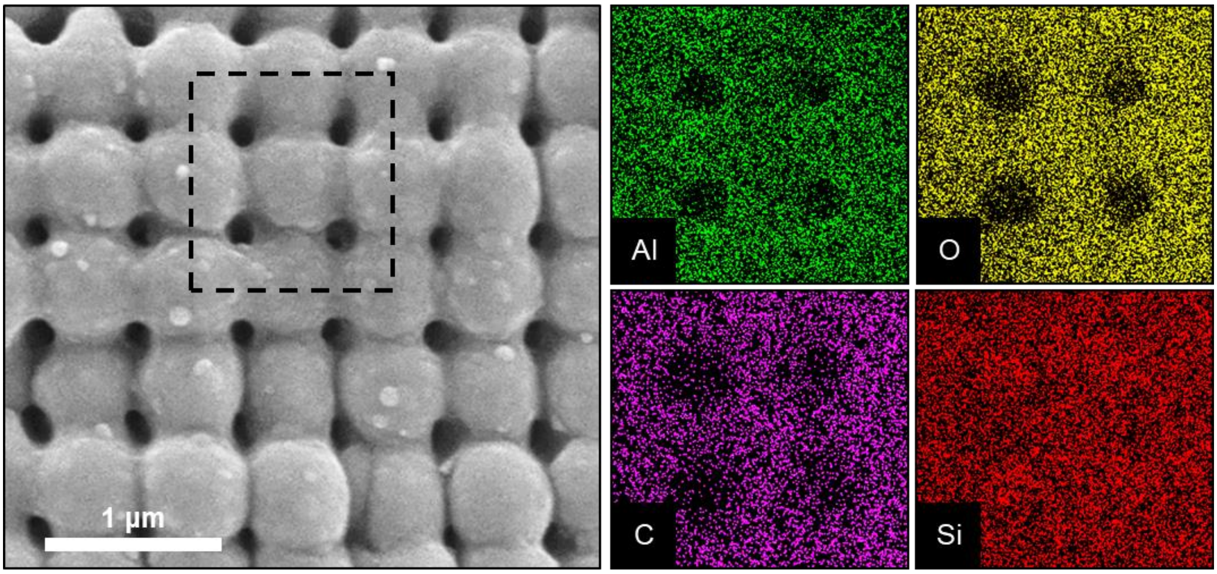

Figure S2. The elemental mapping of the 3D nanocomposite (top-view) after depositing the Al_2O_3 nanoshell on the 3D PDMS.

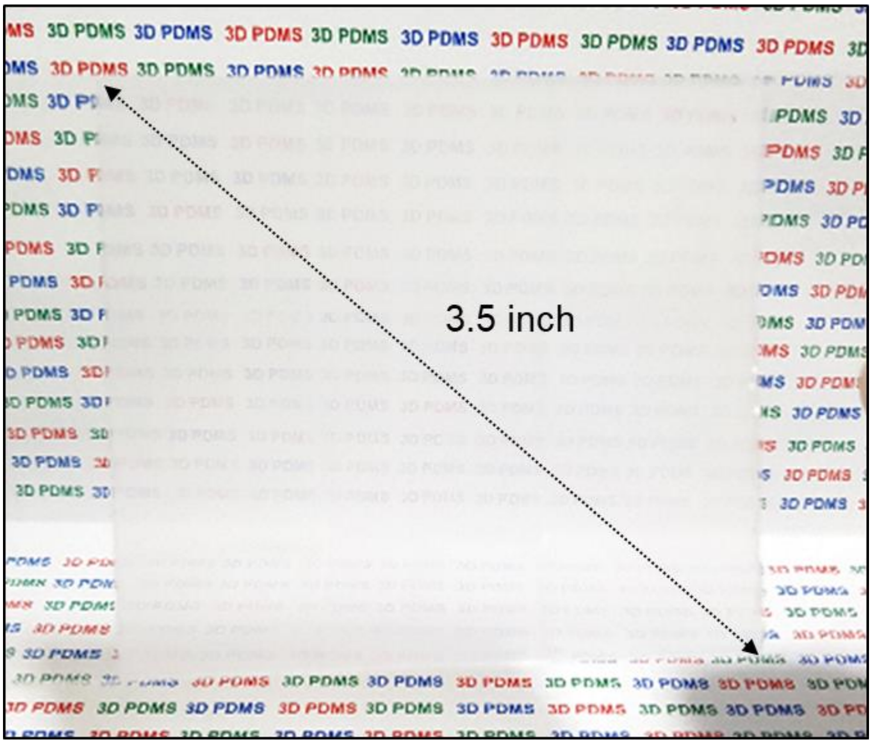

Figure S3. Digital image of the large-area 3D nanocomposite film before index matching by infiltrating the PDMS.

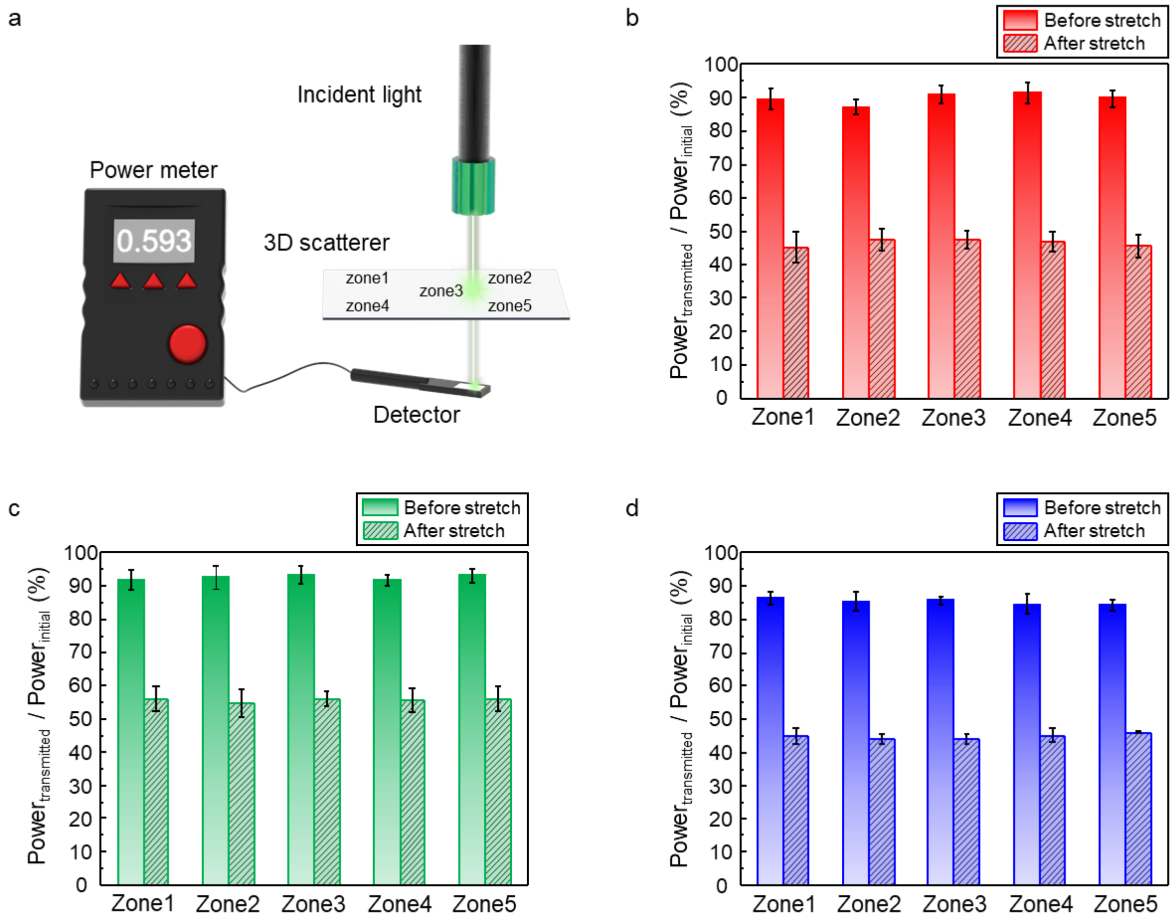

Figure S4. Change in transmitted power through the 3D scatterer in different positions of the film. a) Schematic illustration of the measurement at various positions of 3D scatterer. b) Ratio of transmitted power to initial power for the red light at the corresponding positions before/after stretching. c) that of the green light, and d) that of the blue light.

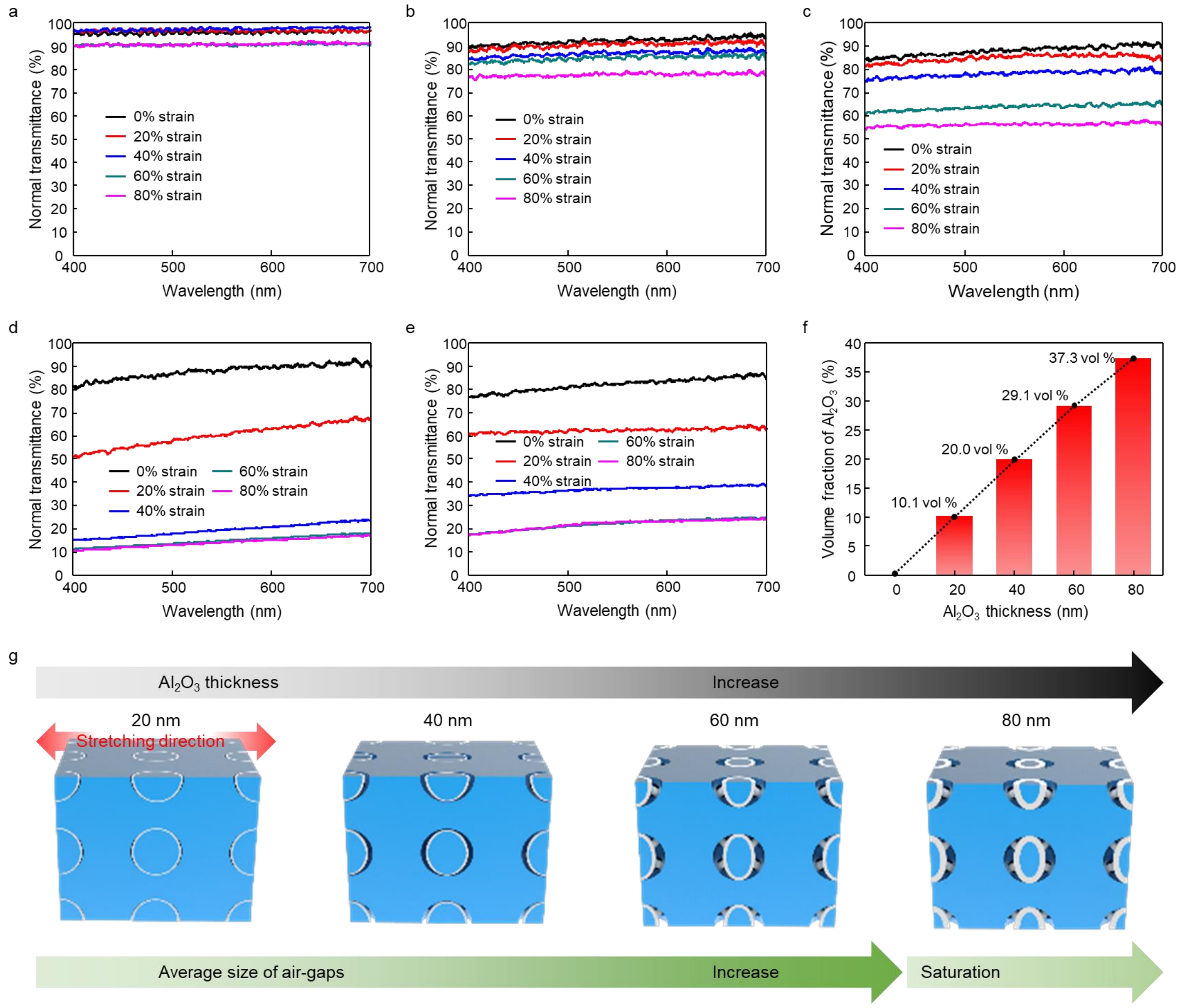

Figure S5. Normal transmittance versus wavelengths of the 3D scatterer with different Al_2O_3 thickness of a) 0 nm, b) 20 nm, c) 40 nm, d) 60 nm, and e) 80 nm, as a function of applied tensile strain. f) Calculated volume fraction of the Al_2O_3 nanoshell. g) Schematic illustrations of the unit cell in 3D scatterer.

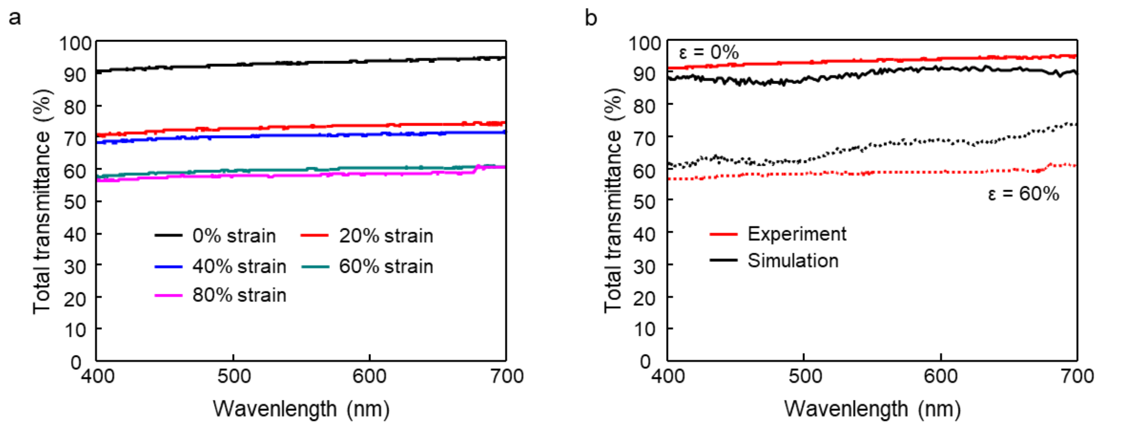

Figure S6. Total transmittance of the 3D scatterer. a) Measured total transmittance versus wavelengths of the 3D scatterer with Al_2O_3 thickness of 60 nm, as a function of tensile strain. b) Comparison of the experimental and simulated results before and after stretching under a strain of 60%.

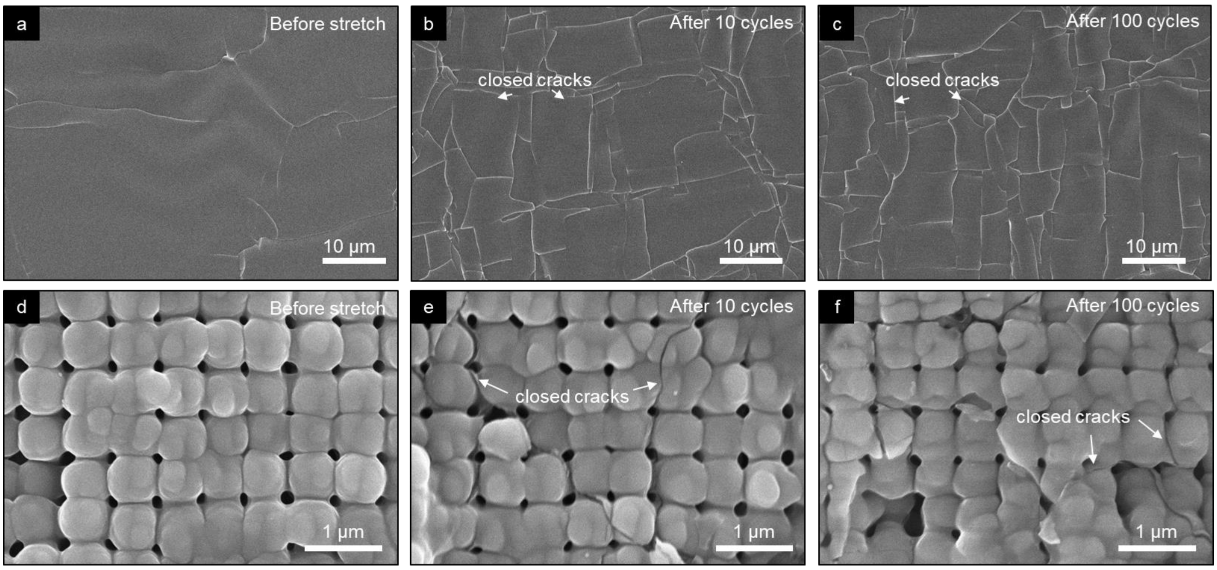

Figure S7. Closing of the strain-induced cracks after releasing the applied strain. a) SEM images of a bilayer structure (a bulk PDMS film with 60 nm thick Al_2O_3 thin layer) before stretching, b) after repeated 10 cycles of stretching and releasing the strain, and c) after 100 times of repeated cycles. d) A SEM image of the 3D nanocomposite (the 3D PDMS with 60 nm thick Al_2O_3) before stretching, e) after repeated 10 cycles of stretching and releasing the strain, and f) after 100 times of repeated cycles.

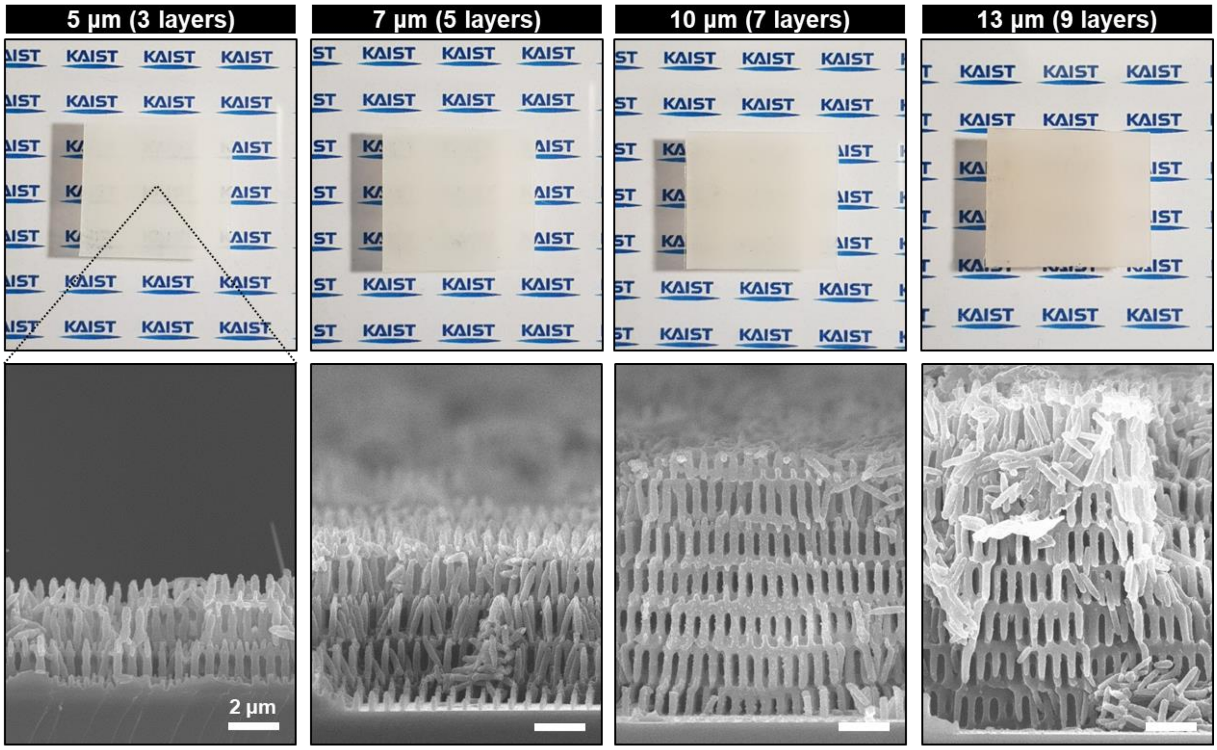

Figure S8. Digital images and the corresponding cross-sectional SEM images of the 3D polymeric templates. The samples with 5 µm thickness (3 scattering layers), 7 µm thickness (5 scattering layers), 10 µm thickness (7 scattering layers), and 13 µm thickness (9 scattering layers), defined by the PnP techniques.

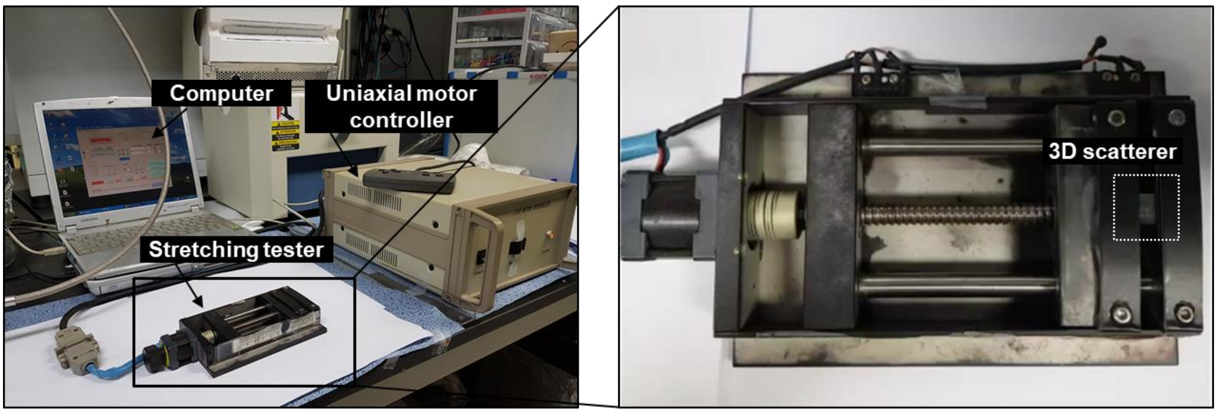

Figure S9. Repeating stretching and releasing cyclic test. Stretching tester connecting to the uniaxial motor controller to relay switch applying/releasing tensile strain to investigate cyclic properties of the 3D scatterer.

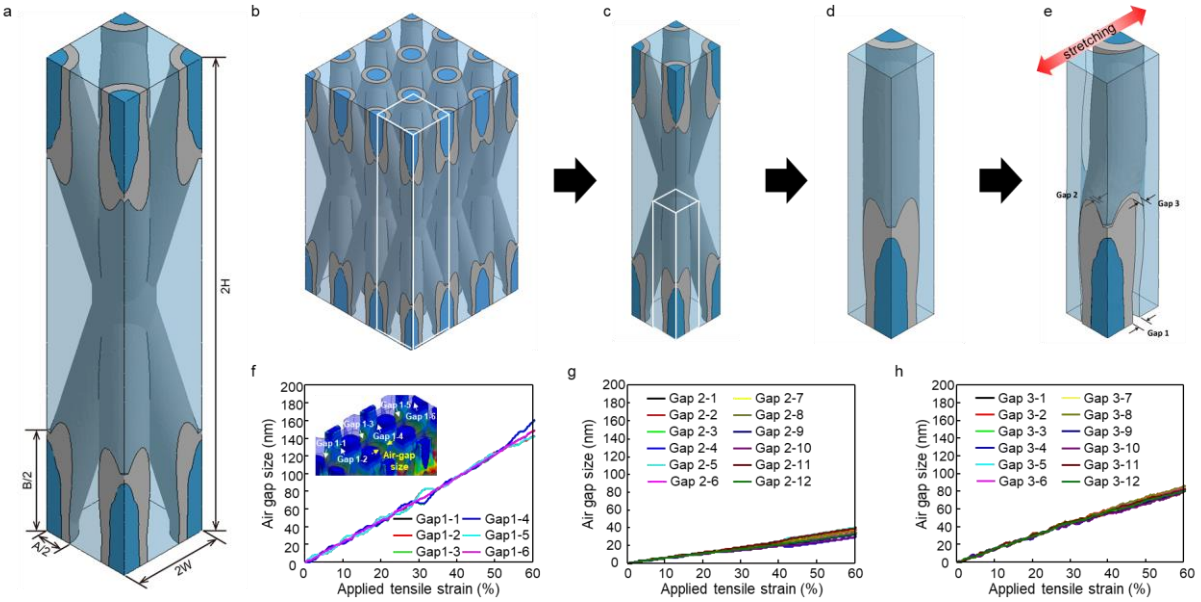

Figure S10. Unit cells of the 3D scatterer and size plots of the generated gaps under stretching. a) The unit cell consists of ellipsoids with an equatorial and polar diameter of A and B (A/2 = 175 nm and B/2 = 660 nm), respectively, and the filling part as an inverse structure. The ellipsoids are packed in a body-centered tetragonal structure with the base of 2W×2W and height of 2H (2W = 600 nm and 2H = 3.1 μm). b) 9 unit cells, c) 1 unit cell, d) 1/8 unit cell, and e) stretched state of 1/8 unit cell of the 3D scatterers, respectively. f) The size plot for linearly evolving gap 1, g) that of the gap 2, and h) that of gap 3 in the successive 3 stretched unit cells.

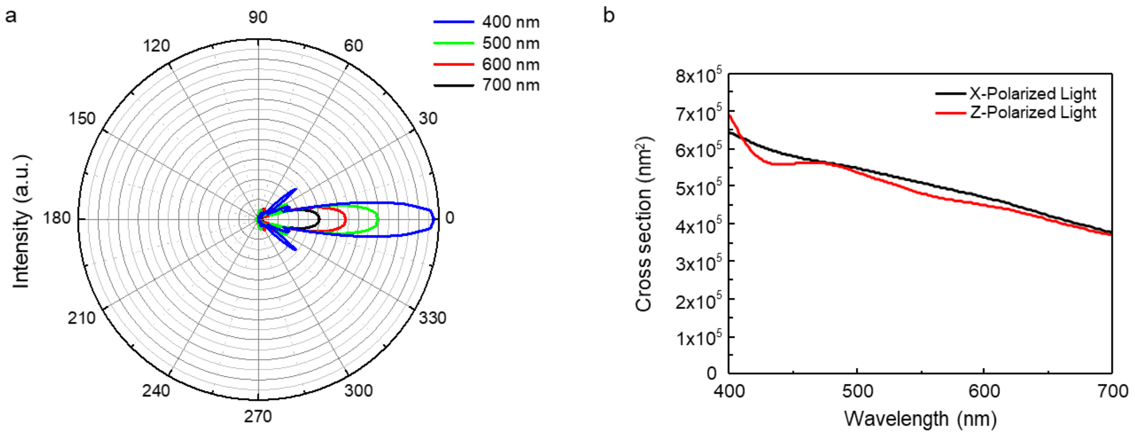

Figure S11. Scattering simulations of the ASZ in 60 % stretched 3D scatterer. a) Scattering phase functions for 4 different incident lights (wavelength of 400 nm, 500 nm, 600 nm, and 700 nm, respectively). b) Simulated cross section area of the 60 % stretched unit cell of the 3D scatterer.

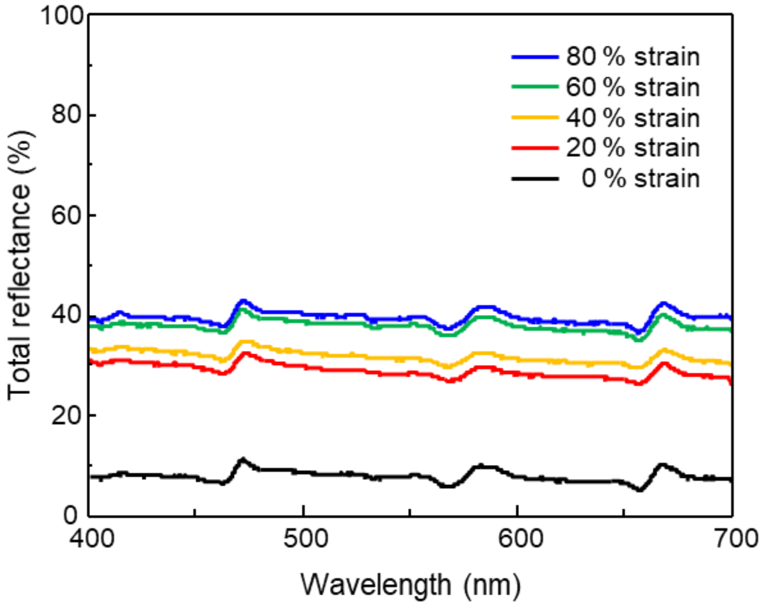

Figure S12. Total reflectance plot of the 3D scatterer with the Al_2O_3 thickness of 60 nm and the film thickness of 13 μm as a function of tensile strain.

Supporting References

[1] Wu, R. B., Itoh, T. *IEEE Transactions on Antennas and Propagation* **1997**, 45, 1302.

[2] Cavalieri, F. J., Cardona, A. *Latin American Appl Research* **2012**, 42, 281.

[3] Na, Y. E., Shin, D., Kim, K., Ahn, C., Jeon, S., Jang, D. *Small* **2018**, 14, 1802239.

[4] Jeon, S., Park, J.-U., Cirelli, R., Yang, S., Heitzman, C. E., Braun, P. V., Kenis, P. J. A., Rogers, J. A. *Proc. Natl. Acad. Sci. U. S. A.* **2004**, 101, 12428.

[5] Ullal, C. K., Maldovan, M., Wohlgemuth, M., Thomas, E. L., White, C. A., Yang, S. *J. Opt. Soc. Am. A* **2003**, 20, 948.
